# Functional Mitral Valve Regurgitation: Mitral Valve Repair or Replacement? Our “Road Map” for the Appropriate Strategy

**DOI:** 10.3390/jcm13113264

**Published:** 2024-05-31

**Authors:** Konstantinos Sideris, Melchior Burri, Antonia Mayr, Stephanie Voss, Keti Vitanova, Anatol Prinzing, Bernhard Voss, Andrea Amabile, Arnar Geirsson, Markus Krane, Ralf Guenzinger

**Affiliations:** 1 Department of Cardiovascular Surgery, Institute Insure, German Heart Center Munich, School of Medicine & Health, Technical University of Munich, Lazarettstrasse 36, 80636 Munich, Germany; sideris@dhm.mhn.de (K.S.); prinzing@dhm.mhn.de (A.P.);; 2 Division of Cardiac Surgery, Department of Surgery, Yale School of Medicine, New Haven, CT 06510, USA; amabile.andrea@gmail.com; 3 Division of Cardiac Surgery, Department of Surgery, Columbia University, New York City, NY 10032, USA; 4 DZHK (German Center for Cardiovascular Research)—Partner Site Munich Heart Alliance, 80636 Munich, Germany

**Keywords:** functional mitral regurgitation, mitral valve repair, mitral valve replacement

## Abstract

**Objectives:** The optimal surgical approach for the treatment of functional mitral regurgitation (FMR) remains controversial. Current guidelines suggest that the surgical approach has to be tailored to the individual patient. The aim of the present study was to clarify further aspects of this tailored treatment. **Methods:** From 01/2006 to 12/2015, 390 patients underwent mitral valve (MV) surgery for FMR (ischemic *n* = 241, non-ischemic *n* = 149) at our institution. A regression analysis was used to determine the effect of MV repair or replacement on survival. The patients were analyzed according to the etiology of the MR (ischemic or non-ischemic), different age groups (<65 years, 65–75 years, and >75 years), LV function, and LV dimensions, as well as the underlying heart rhythm. **Results:** The overall survival rates for the repair group at 1, 5, and 8 years were 86.1 ± 1.9%, 70.6 ± 2.6%, and 55.1 ± 3.1%, respectively. For the same intervals, the survival rates in patients who underwent MV replacement were 75.9 ± 4.5%, 58.6 ± 5.4%, and 40.9 ± 6.4%, respectively (*p* = 0.003). Patients younger than 65 years, with an ischemic etiology of FMR, poor ejection fraction (<30%), severe dilatation of left ventricle (LVEDD > 60mm), and presence of atrial fibrillation had significantly higher mortality rates after MV replacement (HR, 3.0; CI, 1.3–6.9; *p* = 0.007). Patients between 65 and 75 years of age had a higher risk of death when undergoing mitral valve replacement (HR, 1.7; CI, 1.0–2.8; *p* = 0.04). In patients older than 75 years, the surgical approach (MV repair or replacement) had no effect on postoperative survival (HR, 0.8; CI, 0.4–1.3; *p* = 0.003). **Conclusions:** Our data demonstrate that, in patients younger than 65 years, the treatment of choice for FMR should be MV repair. This advantage was even more evident in patients with an ischemic origin of MR, a poor ejection fraction, a severe LV dilatation, and atrial fibrillation.

## 1. Introduction

The optimal surgical approach for the treatment of FMR remains a controversial topic. In contrast to degenerative mitral regurgitation, in which mitral valve repair is highly recommended, current guidelines for FMR suggest that the surgical approach has to be tailored to the individual patient [1]. Depending on the underlying MV pathology (ischemic or non-ischemic), the stage of the left ventricular disease, or the presence of predictors for repair failure, either repair or replacement of the MV can be performed [1,2,3,4,5,6,7,8,9]. Despite the clinical evidence on this topic, there is still room for interpretation. In the description of the etiology, the term functional mitral regurgitation or secondary mitral regurgitation is usually used. However, each study investigates a specific underlying condition. Furthermore, additional factors such as age are often not taken into account. In fact, in real settings, the distinction between the different causes of functional mitral regurgitation is not clear enough, so that often the surgical approach is left to the surgeon’s discretion. There is therefore a need to simplify the whole process and provide a decision-making tool for the surgeon. The aim of the present study was to identify which subgroup of patients would benefit from mitral valve repair over replacement, to enable an individually tailored treatment.

## 2. Materials and Methods

### 2.1. Study Design and Population

All patients who underwent MV surgery for FMR between January 2006 and December 2015 at our institution were included in the present study. From a total of 390 patients, 303 (77.7%) patients underwent MV repair and 87 (22.3%) underwent MV replacement. Preoperative, periprocedural, and postoperative data were prospectively collected in a dedicated database and retrospectively analyzed. The study was approved by the local institutional Ethics Committee (approval reference number: 564/16 S, 14 December 2016). The baseline data are presented in Table 1. The comparison of surgical techniques in the different age groups is provided in the Supplementary Table S1. All included patients had at least moderate FMR, which was confirmed by preoperative transthoracic, preoperative transesophageal, or intraoperative transesophageal echocardiography. The etiology of FMR was ischemic in 241 patients (61.8%) and non-ischemic in 149 patients (38.2%). Patients with acute myocardial infarction, ventricular septal defect, papillary muscle rupture, ventricular aneurysm, severe right ventricular dysfunction, and multiple organ failure were excluded from the study. The classification of FMR into ischemic and non-ischemic was in accordance with the Carpentier Classification for mitral regurgitation and was consistent with Type I or IIIb.

### 2.2. Study Objective

This study compared MV repair and MV replacement patients with FMR. The aim was to identify factors that could affect the postoperative survival in the respective groups.

### 2.3. Surgical Procedure

Our policy for the treatment of functional mitral regurgitation has been based on the current data on the subject. The initial enthusiasm about the superiority of mitral valve repair was attenuated by the publication of data from prospective randomized trials showing that both mitral valve replacement and repairs were equal. Concurrently, there have been developments in the field of mitral valve repair. The introduction of 3-dimensional annuloplasty rings specifically designed for FMR was an important step for successful interventions with durable results. These factors were included in our decision making on whether to replace or repair the mitral valve. If there were echocardiographic predictors of repair failure, such as higher mitral annular diameter and higher tethering area or unfavorable anatomical conditions for MV repair, we preferred MV replacement. In the case of MV replacement, the aim was always to preserve the subvalvular apparatus as far as possible. Various types of rings were initially used for MV repair, which were gradually replaced by 3-dimensional, closed, and rigid annuloplasty rings.

### 2.4. Statistics

Normally distributed continuous variables were presented as mean ± standard deviation (SD). Categorical variables were presented as frequencies (%). Student’s *t*-test, chi-square test, or Fisher’s exact test were used to compare the preoperative and procedural data, as appropriate. Overall survival was analyzed with the Kaplan–Meier method and compared between groups using a log-rank test. The Hazard Ratio (HR) was determined by using Cox regression. To determine the effect of mitral valve repair in comparison to mitral valve replacement for different age groups, and to adjust for covariates, we divided the patients in three subgroups >65 years, 65–75 years, and <75 years. Within those subgroups, the effect of repair versus replacement was investigated using a univariate Cox regression model for the complete subgroups and for patients with different characteristics (ischemic or non-ischemic origin, left ventricular ejection fraction, left ventricular dimensions, and the presence of atrial fibrillation). These covariates were chosen prior to any analysis based on their assumed clinical relevance. Statistical analysis was performed with IBM SPSS 22 (SPSS Inc., Chicago, IL, USA) and R (version 3.5.2; R Foundation for Statistical Computing, Vienna, Austria).

## 3. Results

The mean follow-up time of the entire cohort was 5.4 ± 3.6 years. The mean follow-up in the patients with ischemic etiology was 5.4 ± 3.6 years, and in the non-ischemic group, it was 5.8 ± 3.5 years. Of the patients who were alive at the last follow-up (*n* = 193), 90.7% had a complete follow-up with echocardiographic studies and functional status. In 18 patients (9.3%), only the survival status could be determined.

Table 2 summarizes the periprocedural data. MV replacement was performed in 87 patients, and 303 patients underwent MV repair. From those, 124 patients received a 2-dimensional annuloplasty ring, and 176 patients underwent MV repair by using a 3-dimensional annuloplasty ring.

### 3.1. Reoperations and Freedom from Residual Mitral Regurgitation

During the follow-up period, nine patients (2.3%) required reoperation. In seven patients, the reason was recurrent MR. The reoperation cases are summarized in Table 3. At the latest follow-up, we observed no patients with more than moderate MR.

### 3.2. Operative Mortality and Overall Survival

**Figure 1:**
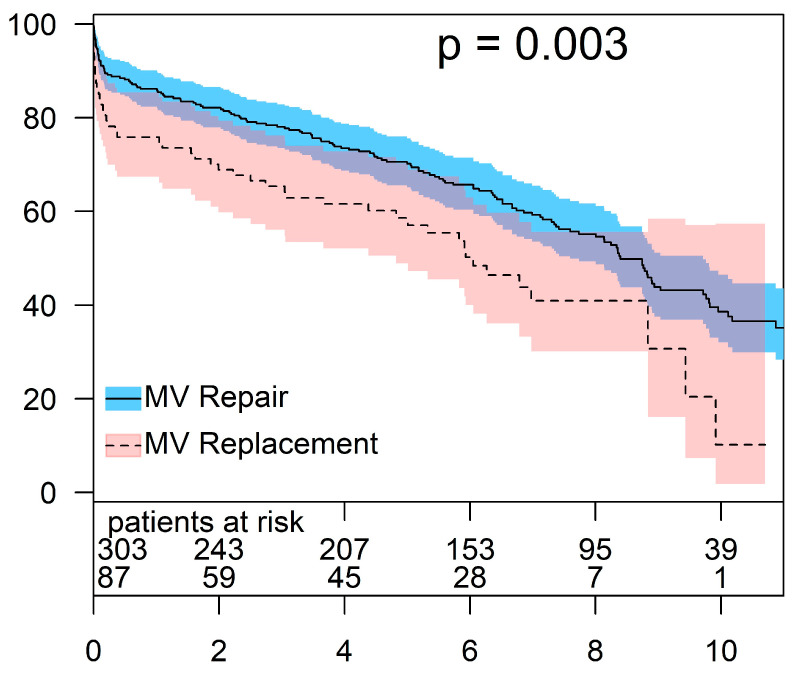
Overall survival in MV repair vs. MV replacement.

The overall 30-day mortality rate was 8.5% (*n* = 33), and it was significantly lower in patients who underwent MV repair compared to those who underwent MV replacement (8.5% vs. 14.9%, *p* = 0.026). At the latest follow-up, 225 patients were alive (57.7%). Amongst those, 182 patients were in the repair group, and 43 patients were in the replacement group. The overall survival was significantly lower in patients who underwent MV replacement for FMR. The estimated 1-, 5-, and 8-year survival rates for the repair group were 86.1 ± 2.0%, 70.2 ± 2.7%, and 55.1 ± 3.1%, respectively. For the same intervals, the survival rates in patients who underwent MV replacement were 75.9 ± 4.6, 58.6 ± 5.4, and 40.9 ± 6.4%, respectively (*p* = 0.003) (Figure 1).

Because of the different etiology of FMR, we further compared the two approaches (MV repair or replacement) based on the underlying pathology (ischemic and non-ischemic).

### 3.3. Survival in Patients with Ischemic Functional Mitral Regurgitation

**Figure 2:**
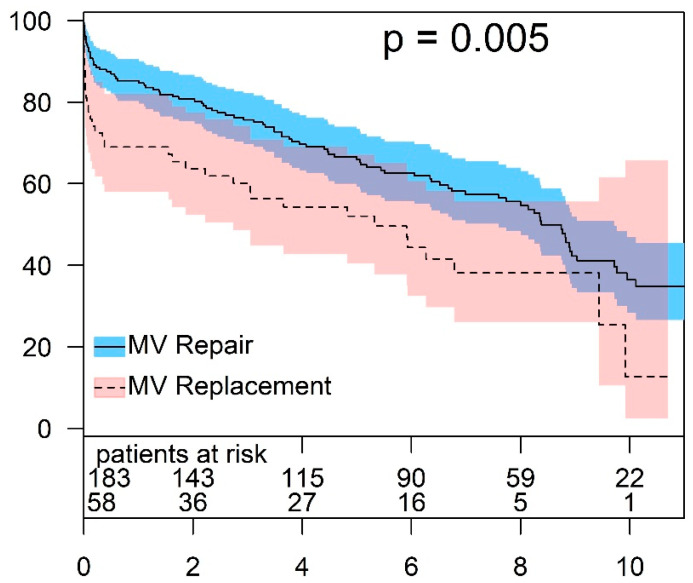
Survival after treatment of ischemic MV disease (MV repair vs. MV replacement).

The 30-day mortality in patients undergoing MV surgery for FMR with ischemic etiology was 10.4% (25/241). The 30-day mortality in the MV repair and in the MV replacement group was 6.6% (12/183) and 22.4% (13/58), respectively (*p* = 0.008). Patients with an ischemic origin of FMR showed a higher survival rate after MV repair compared to those who received a MV replacement. The estimated 1-, 5-, and 8-year survival rates for the repair group were 85.5 ± 2.6%, 65.9 ± 3.6%, and 55.6 ± 4.0%, respectively. For the same intervals, the survival rates in patients who underwent MV replacement were 69.0 ± 6.1%, 52.0 ± 6.7%, and 38.1 ± 7.3%, respectively (*p* = 0.005) (Figure 2).

### 3.4. Survival in Patients with Non-Ischemic Functional Mitral Regurgitation

**Figure 3:**
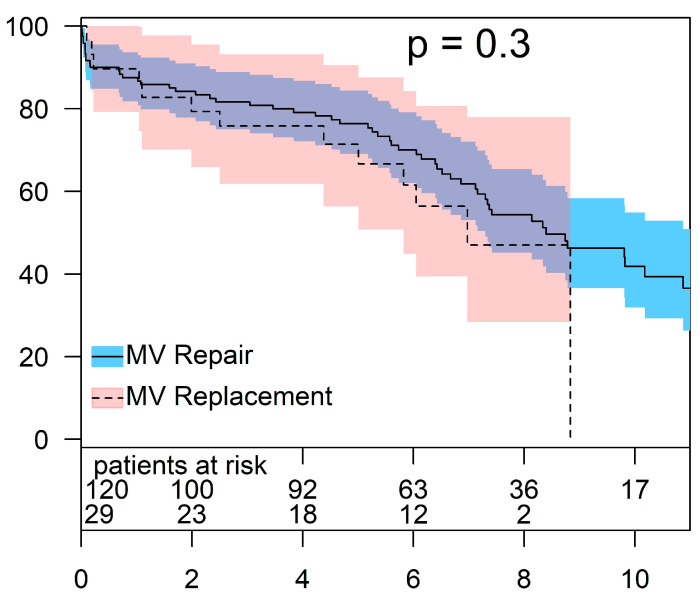
Survival after treatment of non-ischemic MV disease (MV repair vs. MV replacement).

Patients with non-ischemic origin of MR had an overall 30-day mortality of 5.4% (8/149 patients). The 30-day mortality for patients undergoing MV repair and MV replacement was 6.7% (8/120) und 0% (0/29), respectively. In this cohort, the overall survival in patients who underwent MV repair was 87.5 ± 3.0%, 76.4 ± 3.9%, and 54.3 ± 5.1% at 1, 5, and 8 years, respectively. For the same intervals, the survival rates in patients who underwent MV replacement were 89.7 ± 5.7%, 71.4 ± 8.6%, and 47.0 ± 12.1%, respectively (*p* = 0.3) (Figure 3).

### 3.5. Effect of the Surgical Approach on Survival within Patients’ Age Subgroups

**Figure 4:**
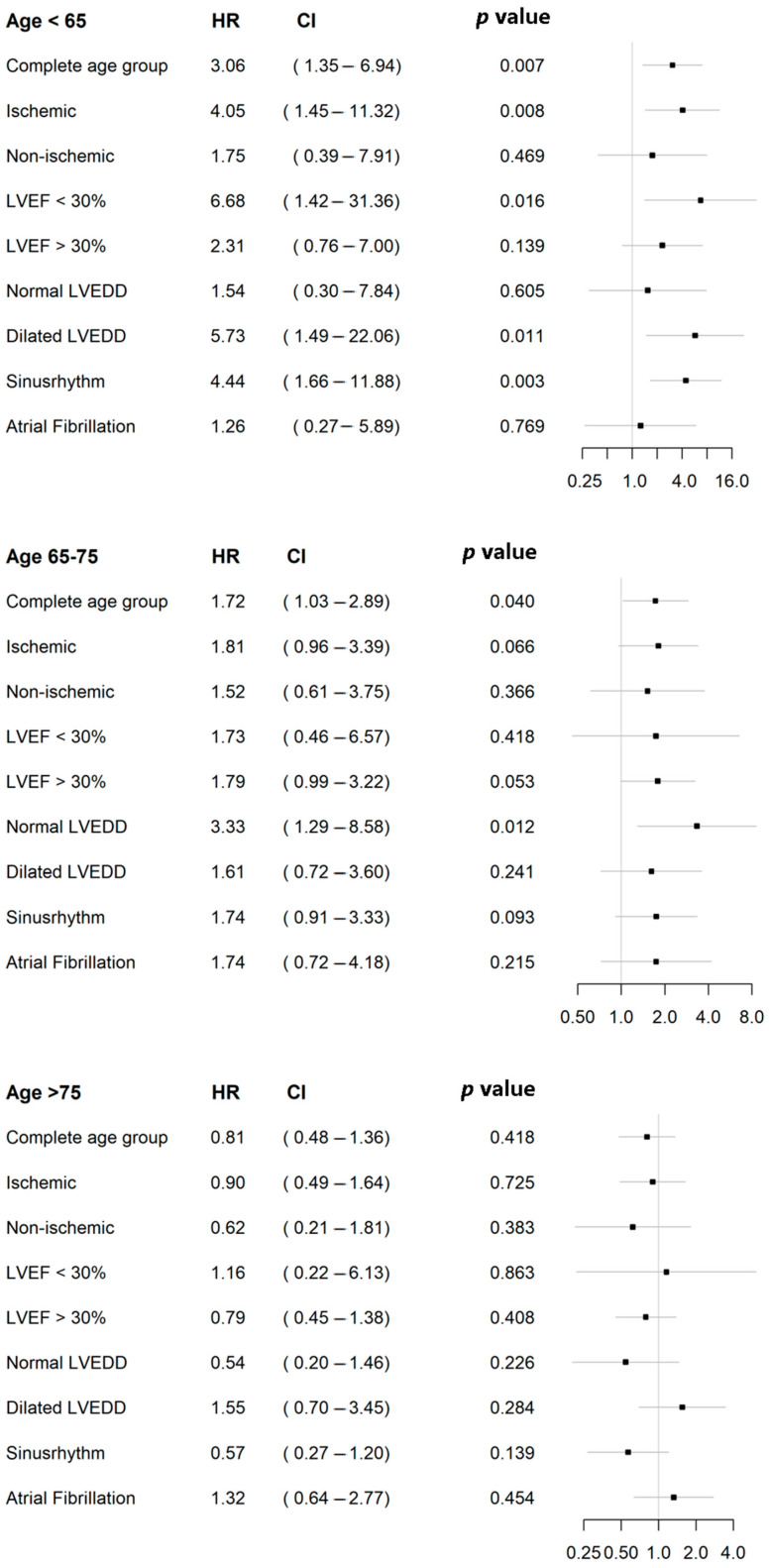
Effect of surgical approach within patients‘ age subgroups.

Cox regression was used to determine the effect of MV repair or replacement on survival. Patients were analyzed according to the etiology of the MR (ischemic or non-ischemic), the age groups (<65 years, 65–75 years, and >75 years), the LV function, the LV dimensions, and the underlying heart rhythm (Figure 4).

#### 3.5.1. Risk Factors for Mortality in Younger Patients (<65 Years)

Patients younger than 65 years with ischemic MR, poor ejection fraction (EF < 30%), severe dilatation of the left ventricle (LVEDD > 60 mm), and the presence of atrial fibrillation showed a significantly higher risk for mortality after MV replacement (Figure 4). Overall, patients younger than 65 years had a three-fold higher risk of death if they received mitral valve replacement instead of mitral valve repair (HR, 3.0; CI, 1.3–6.9; *p* = 0.007).

#### 3.5.2. Risk Factors for Mortality in Elderly Patients (65–75 Years)

Patients between 65 and 75 years had a higher risk for mortality after MV replacement (HR, 1.7; CI, 1.0–2.8; *p* = 0.04). However, we could not demonstrate that this was influenced by specific subgroups, except for patients with normal ventricular dimensions who underwent MV replacement. These patients had an increased risk of postoperative mortality when receiving an MV replacement (HR, 2.8; CI, 1.1–6.6; *p* = 0.02) (Figure 4).

#### 3.5.3. Risk Factors for Mortality in Older Patients (>75 Years)

In patients older than 75 years, the surgical approach (MV repair or replacement) had no effect on the postoperative survival (HR, 0.8; CI, 0.4–1.3; *p* = 0.003). No significant factors affecting survival were identified. (Figure 4).

### 3.6. Survival in Age Groups

**Figure 5:**
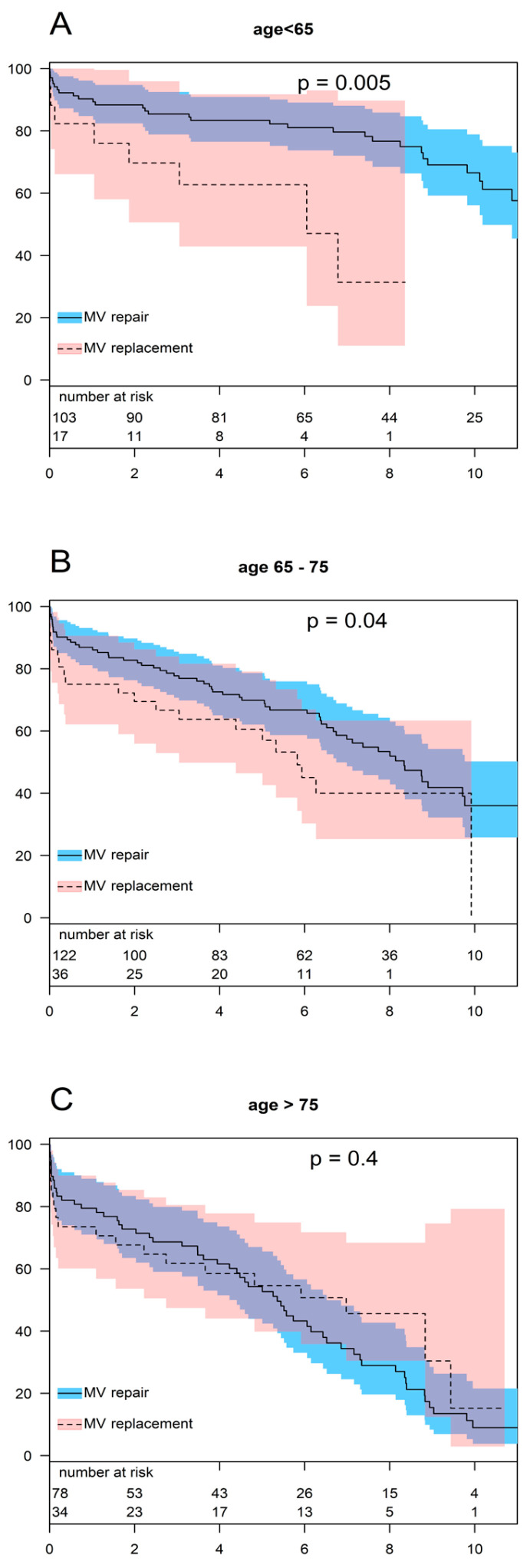
Survival in age groups. (Survival rates of patients aged, (**A**): <65; (**B**): 65–75; (**C**): >75).

The findings from our regression model showed the superiority of MV repair in patients younger than 65 years. Figure 5 shows a comparison of the survival rates for MV repairs and MV replacements stratified by age. Survival was significantly higher in younger patients who underwent MV repair compared to those who underwent MV replacement (*p* = 0.005). After 1, 5, and 8 years, the survival rates in the repair group were 90.3 ± 2.5%, 83.4 ± 3.6%, and 76.7 ± 4.6%, respectively. For the same intervals, the survival rates of patients who underwent MV replacement were 82.4 ± 9.2%, 62.7 ± 12.1%, and 31.4 ± 16.8%, respectively.

The advantage of repair that we detected in younger patients was reduced in elderly patients (65–75 years). Nevertheless, MV repair was slightly superior in this group as well. The estimated 1-, 5-, and 8-year survival rates for the repair group were 86.9 ± 3.0%, 69.8 ± 4.2%, and 53.4 ± 5.0%, and for the replacement group, they were 75.0 ± 7.2%, 60.6 ± 8.2%, and 40.0 ± 9.3%, respectively (*p* = 0.037).

In patients over 75 years of age, the survival rates after MV repair or MV replacement were equal. After 1, 5, and 8 years, the survival rates in the repair group were 79.4 ± 4.5%, 54.3 ± 5.8%, and 28.9 ± 5.7%, respectively. For the same intervals, the survival rates in patients who underwent MV replacement were 73.5 ± 7.5%, 54.6 ± 8.7%, and 45.6 ± 9.4%, respectively (*p* = 0.415).

## 4. Discussion

Our results demonstrated that in the case of functional MR, MV repair compares favorably to MV replacement in patients younger than 65 years of age. This was even more evident in patients < 65 years with an ischemic origin of MR, a poor ejection fraction, a severe LV dilatation, and atrial fibrillation. In addition, we observed a low reoperation rate due to recurrent MR in patients undergoing MV repair for functional MR. At latest follow-up, no patient presented with more than moderate mitral regurgitation. In previous studies from our institution, we have demonstrated that MV repair for functional MR was associated with low or no recurrent MR [10,11,12]. These findings resonate with prior evidence suggesting that high rates of recurrent MR may attenuate the potential benefits of mitral valve repair [2,3,13,14,15].

Despite concerns that the presumed high MR recurrence rate has a negative impact on survival, several studies reported a higher survival rate in patients who underwent MV repair for functional MR compared to those who underwent MV replacement [3,9]. This was explained by the assumption that patients undergoing replacement were frequently older and had more comorbidities than those with MV repair [3]. We therefore compared both procedures stratified by age groups. Thereby, we could demonstrate that not only age but also additional factors (such as LV dilatation, LV function, and the origin of mitral regurgitation) had a significant impact on survival after MV repair or MV replacement.

Formerly, MV repair was considered to be the standard treatment for patients with functional MR [9,14,16,17,18,19]. After the publication of selected data from randomized trials, the interest has increasingly shifted towards mitral valve replacement, initiating a new view on the treatment of functional MR [2,20]. These trials examined the outcome of patients after MV repair or MV replacement in case of ischemic MR. At 2 years, there was no significant difference in mortality between the two groups. These studies were well designed with important results but addressed a very specific spectrum of functional MR. We are strongly convinced that a patient’s age at the time of surgery has to be taken into account in the decision-making process, and this aspect may have not been addressed in the trials.

An interesting observation in our results was the reoperation rate and the number of patients with more than moderate MR at the latest follow-up. Regarding the reoperation rate, our results were absolutely in line with those from prospective randomized trials. Acker et al. showed a reoperation rate of 2.3% after repair [2]. In our cohort, the overall reoperation rate was 2.3%. However, in contrast to the study mentioned above, we were able to show that we had no patient with more than moderate MR at the time of the last FU. Certainly, it has to be taken into account that the retrospective nature of the study can also lead to a possible bias. However, the comparison shows that our results are valid.

The treatment of functional MR is still a matter of debate, in spite of the recent evidence integrated into the new guidelines for the management of valvular heart disease [1] The more general term “tailored treatment” was newly introduced, and this generic terminology may pave the way for generic and contradictory interpretations. In order to attempt to close this gap, we leveraged our findings to create a roadmap for the treatment of functional MR.

### “Roadmap” for the Surgical Treatment of Functional MR

Patients younger than 65 years of age have a three-fold higher risk of death in the case of MV replacement compared to MV repair. In the same group of age, MV repair is superior in patients with ischemic MR. Likewise, patients with poor LV function and significant left ventricular dilatation benefit from MV repair in terms of the long-term survival. The superiority of MV repair is also evident in the patients aged 65–75 years, especially in patients with normal ventricular dimensions. Comparing both procedures based on the etiology of the MR, the LV function, and the cardiac rhythm, we found that these factors did not influence survival for the respective procedure. In patients older than 75 years, repair or replacement had no differential effect on postoperative survival. Whether the patient had preserved or poor LV function, normal or enlarged ventricular dimensions, or whether the etiology was ischemic or non-ischemic, either procedure (repair or replacement) was performed without significant differences in survival.

## 5. Conclusions

In summary, patients younger than 65 years with FMR should undergo MV repair. In particular, younger patients with an ischemic etiology of MR, a poor ejection fraction and an enlarged ventricle, have a significantly higher survival rate when receiving an MV repair over replacement. The superiority of MV repair is also evident in patients aged 65–75 years. Patients over 75 years of age show comparable survival rates with either procedure.

## 6. Limitations

The present study has several limitations. First, this is a single-center retrospective study. Due to the controversy surrounding the definition of secondary mitral regurgitation (MR), we decided to use the more practical term “non-ischemic” FMR for patients with MR caused by left ventricular disease (dilatation, abnormal shape, and/or dysfunction) and/or dilatation of the left atrium. Patients in whom coronary artery disease was causally responsible for LV dysfunction and severe MR were referred to as “ischemic” FMR. This simplification of the terminology was an attempt to standardize the different pathologies for the purpose of our analyses; however, it can certainly be challenged. It should be noted that there is probably a selection bias, but this cannot be identified or controlled due to the retrospective nature of the study. Although the groups (repair and replacement) are comparable in the baseline data, we cannot exclude the possibility that MV replacement was performed in patients with poorer preoperative conditions. The data indicate that these patients were certainly older. This could affect the results and thus the comparability of the two groups. Furthermore, the division of patients into subgroups resulted in a smaller number of patients in each analysis. However, due to the relatively high mortality rate during follow-up, the number of events was sufficient to perform a univariate Cox regression model (mortality depending on treatment mode) also within the age groups. The number of reoperations was low and did not allow further analysis of risk factors for reoperations. Finally, echocardiographic follow-up was performed by local experienced cardiologists. The lack of blind review of the echocardiography data may introduce some degree of interpretation bias.

## Figures and Tables

**Table 1:** Baseline characteristics categorized by etiology.

		MV Repair	MV Replacement	*p* Value
All patients		*n* = 303	*n* = 87	
	Female, *n* (%)	124 (41%)	41 (47%)	0.363
	Age (years) ^a^	67 ± 11	71 ± 11	0.008
	LV-EF (%)	45.7 ± 14.7	47.7 ± 14.2	0.254
	Afib, *n* (%)	112 (37%)	33 (37.9%)	0.969
	Creatinin (mg/dL)	1.2 ± 0.6	1.2 ± 0.5	0.585
	LVEDD (mm) ^a^	57.3 ± 8.2	55.5 ± 10.3	0.235
	EuroScore 2 ^a^	6.6 ± 7.9	10.0 ± 11.4	0.011
Non-ischemic FMR		*n* = 120	*n* = 29	
	Female, *n* (%)	62 (51.7%)	20 (69%)	0.141
	Age (years) ^a^	65 ± 13	70 ± 13	0.083
	LV-EF (%)	50.9 ± 13.1	51.7 ± 14.6	0.782
	Afib, *n* (%)	57 (48%)	9 (31%)	0.163
	Creatinin (mg/dL)	1.1 ± 0.5	1.1 ± 0.4	0.950
	LVEDD (mm) ^a^	57.3 ± 9.5	52.9 ± 11.1	0.112
	EuroScore 2 ^a^	4.9 ± 5.6	7.8 ± 7.9	0.081
Ischemic FMR		*n* = 183	*n* = 58	
	Female, *n* (%)	62 (34%)	21 (36%)	0.868
	Age (years) ^a^	69 ± 9	71 ± 10	0.070
	LV-EF (%)	42.4 ± 15	45.7 ± 13.7	0.133
	Afib, *n* (%)	55 (30%)	24 (41%)	0.150
	Creatinin (mg/dL)	1.2 ± 0.6	1.2 ± 0.4	0.611
	LVEDD (mm) ^a^	57.3 ± 7.0	57.0 ± 9.6	0.871
	EuroScore 2 ^a^	7.6 ± 8.9	11.1 ± 12.7	0.061

LV-EF: left ventricular ejection fraction; Afib: atrial fibrillation; MV: mitral valve; FMR: functional mitral regurgitation; ^a^: results are presented as mean ± standard deviation.

**Table 2:** Periprocedural data.

		MV Repair	MV Replacement	*p* Value
All patients		*n* = 303	*n* = 87	
	CPB time (min) ^a^	145.6 ± 56.5	175.4 ± 82.7	0.002
	X-clamp time (min) ^a^	96.4 ± 37.4	112.0 ± 48.4	0.008
	Urgent surgery, *n* (%)	17 (5.6%)	5 (5.8%)	1.000
	Concomitant procedure			
	CABG, *n* (%)	147 (48.5%)	38 (43.7%)	0.500
	Aortic valve surgery, *n* (%)	83 (27.4%)	17 (19.5%)	0.180
	Tricuspid valve surgery, *n* (%)	96 (31.7%)	42 (48.3%)	0.006
	Ablation, *n* (%)	34 (11.2%)	11 (12.6%)	0.861
	Average length of stay (days)	10	14	<0.001
Non-Ischemic FMR		*n* = 120	*n* = 29	
	CPB time (min) ^a^	129.0 ± 57.7	155.0 ± 68.1	0.069
	X-clamp time (min) ^a^	86.2 ± 41.0	100.0 ± 44.2	0.128
	Urgent surgery, *n* (%)	4 (3.3%)	1 (3.5%)	1.000
	Concomitant procedure			
	CABG, *n* (%)	0	0	
	Aortic valve surgery, *n* (%)	46 (38%)	9 (31%)	0.605
	Tricuspid valve surgery, *n* (%)	49 (41%)	18 (62%)	0.064
	Ablation, *n* (%)	22 (18.3%)	3 (10.3%)	0.411
	Average length of stay (days)	9	19	<0.001
Ischemic FMR		*n* = 183	*n* = 58	
	CPB time (min) ^a^	156.0 ± 53.0	186.0 ± 88.	0.019
	X-clamp time (min) ^a^	103.0 ± 33.3	118.0 ± 49.8	0.041
	Urgent surgery, *n* (%)	13 (7.1%)	4 (6.9%)	1.000
	Concomitant procedure			
	CABG, *n* (%)	147 (80.3%)	38 (65.5%)	0.032
	Aortic valve surgery, *n* (%)	37 (20.2%)	8 (13.8%)	0.368
	Tricuspid valve surgery, *n* (%)	47 (25.7%)	24 (41.4%)	0.034
	Ablation, *n* (%)	12 (6.6%)	8 (13.8%)	0.101
	Average length of stay (days)	10	13	0.092

^a^: results are presented as mean ± standard deviation.

**Table 3:** Reoperations.

Patient	Age at Operation ^a^	Etiology of MR	Procedure at MV	Concomitant Procedure	Time to Redo a	Cause of Redo	Procedure Performed
1.	38	non-ischemic	MV repair Medtronic Galloway Future Band 26 mm	None	10.7	Recurrent MR progression of native valve disease	MV replacement Sorin 27 mm mechanical prosthesis
2.	51	non-ischemic	MV repair Geoform Ring 32 mm	None	6.6	Severe heart failure	HTx
3.	78	ischemic	MV replacement Hancock biological prosthesis 29 mm	CABG	1.5	Endocarditis	MV replacement Hancock biological prosthesis 29 mm
4.	70	non-ischemic	MV repair Geoform Ring 30 mm	None	0.50	Recurrent MR ring dehiscence	MV replacement Hancock biological prosthesis 29 mm
5.	70	non-ischemic	MV repair Medtronic Future Band 30 mm	TV repair	0.88	Recurrent MR progression of native valve disease	MV replacement Sorin 27 mm mechanical prosthesis
6.	71	non-ischemic	MV repair Edwards Physio Ring 34 mm	TV repair	0.16	Recurrent MR ring dehiscence	MV replacement Edwards biological prosthesis 29 mm
7.	67	ischemic	MV repair Medtronic Simulus Ring 30 mm	Aortic valve replacement, CABG	0.13	Recurrent MR progression of native valve disease	MV replacement Hancock biological prosthesis 31 mm
8.	65	ischemic	MV repair Edwards Physio Ring 34 mm	None	0.54	Recurrent MR ring dehiscence	MV replacement Edwards biological prosthesis 33 mm
9.	66	non-ischemic	MV repair Medtronic Colvin Galloway Future Ring 34 mm	Ablation, LAA occlusion	0.02	Recurrent MR ring dehiscence	MV replacement Mosaic biological prosthesis 29 mm

^a^: years.

## Data Availability

The data presented in this study are available on reasonable request from the corresponding author.

## Supplementary Materials

The following supporting information can be downloaded at: https://www.mdpi.com/article/10.3390/jcm13113264/s1, Supplementary Table S1: Baseline characteristics and procedural data categorized by age group.
