# Supplementary material for: Functional Mitral Valve Regurgitation: Mitral Valve Repair or Replacement? Our “Road Map” for the Appropriate Strategy
Source: J Clin Med. 2024 May 31;13(11):3264. doi: 10.3390/jcm13113264 (PMC11172680; doi:10.3390/jcm13113264)
Supplement: Supplementary file 1 [file jcm-13-03264-s001.zip › jcm-2918205-supplementary.pdf]

Supplementary Table S1: Baseline characteristics and procedural data categorized by age group.

		MV Repair	MV Replacement	*p*-Value
**age < 65**		**n=103**	**n=17**	
	Female, n (%)	33 (32.0%)	6 (35.3%)	0.786
	Age (years)[a]	54±9	54±13	0.828
	LV-EF (%)	45±15	44±15	0.930
	Afib, n (%)	26 (26%)	5 (29%)	0.771
	Creatinin (mg/dl)	1.07±0.52	1.18±0.42	0.344
	CABG (n, %)	42(41%)	6 (35%)	0.792
	TV procedure, n (%)	22 (21%)	7 (41%)	0.122
	AV procedure, n (%)	29 (28%)	2 (12%)	0.232
	Afib ablation (n, %)	10 (10%)	2 (12%)	0.678
**age 65-75**		**n=122**	**n=36**	
	Female (n, %)	51(41.8%)	16(44.4%)	0.849
	Age (years)[a]	70±3	71±3	0.128
	LV-EF (%)	44±16	49±15	0.099
	Afib (n, %)	69(58%)	23(66%)	0.440
	Creatinin (mg/dl)	1.15±0.43	1.26±0.49	0.248
	CABG (n, %)	62(51%)	14(39%)	0.256
	TV procedure (n, %)	42(34%)	23(64%)	0.002
	AV procedure (n, %)	33(27%)	9(25%)	1.000
	Afib ablation (n, %)	15(12%)	4(11%)	1.000
**age > 75**		**n=78**	**n=34**	
	Female (n, %)	40(51.3%)	19(55.9%)	0.685
	Age (years)[a]	79±3	79±3	0.831
	LV-EF (%)	46±15	46±14	0.988
	Afib (n, %)	38(51%)	17(53%)	0.836
	Creatinin (mg/dl)	1.37±0.74	1.18±0.43	0.092
	CABG (n, %)	43(55%)	18(53%)	0.840
	TV procedure (n, %)	32(41%)	12(35%)	0.675
	AV procedure (n, %)	21(27%)	6(18%)	0.344
	Afib ablation (n, %)	9(12%)	5(15%)	0.757
